# Health Justice Partnership: An Opportunity to Respond to Childhood Adversity

**DOI:** 10.5334/ijic.8917

**Published:** 2025-03-25

**Authors:** Sarah Loveday, Suzie Forell, Rebecca Bosward, Lingling Chen, Leanne N. Constable, Wilhelmina Ebbett, Ashraful Kabir, Hueiming Liu, Alexandra Preddy, Natalie White, Harriet Hiscock

**Affiliations:** 1 Department of Paediatrics, University of Melbourne, Parkville, Victoria, Australia; 2 Health Services, Murdoch Children’s Research Institute, Melbourne, Victoria, Australia; 3 Health Justice Australia, Darlinghurst, New South Wales, Australia; 4 Sydney Local Health District, Sydney, New South Wales, Australia; 5 The George Institute for Global Health, Camperdown, New South Wales, Australia; 6 Centre for Community Child Health, The Royal Children’s Hospital, Melbourne, Victoria, Australia

**Keywords:** health justice partnership, childhood adversity, integrated care, community

## Abstract

**Background:**

Health justice partnerships (HJP) embed legal support into health care teams to address patient unmet legal needs. Families experiencing adversity are likely to have multiple legal needs yet are unlikely to seek legal assistance. Implementing a HJP within an integrated health and social care hub may improve outcomes for families by addressing adversity related to unmet legal need.

**Methods:**

A mixed-method evaluation of new HJPs was conducted across two sites in Australia. Process data were collected regarding the use of the legal services. Qualitative data explored the experience of legal support.

**Results:**

Forty caregivers were referred across two sites with 38 seen over the first 12 months. Caregivers had an average 2.5 legal issues each; 65% of referrals were for family violence and family law matters, 31% were for civil law matters and 4% were for criminal matters. Qualitative data demonstrated the value of HJP to increase practitioner confidence and understanding of legal support pathways while empowering caregivers to access legal support.

**Conclusions:**

These HJPs improved access to legal support for families experiencing adversity and enabled early intervention. Improved outcomes from HJP depends upon the level of investment from each partner and the level of assistance the HJP can provide.

## Introduction

Childhood adversity encompasses a range of traumatic or stressful events that occur in childhood such as child abuse and neglect, parental mental health disorders, family violence, parental drug abuse, and community violence and has been recognised as a significant contributor to poor mental and physical health in later life [1]. Adversities tend to cluster in families with higher number of adversities associated with poorer outcomes [2]. Many childhood adverse experiences are associated with legal problems e.g. family law, discrimination, tenancy issues, fines and debts, however, often these legal issues are under recognised.

In an Australia-wide survey of legal needs almost half of 20,000 adults surveyed had at least one legal issue, with greater unmet legal needs in individuals who were unemployed, in social housing or single parents [3]. Similarly to childhood adversity, legal issues cluster with 9% of people surveyed experiencing 65% of the legal issues [3]. Individuals with low incomes are likely to have multiple legal needs however are unlikely to access legal assistance [4].

Common barriers to seeking help for legal issues include not recognising that the issue has a legal solution, not knowing how to address the issue, concerns about the stress and repercussions of raising the issue and the accessibility and cost of legal help [3,5,6]. From studies in the USA and UK prior to establishing Health Justice Partnerships (HJPs), rates of legal assistance were low with between 66–85% of people not accessing legal advice or support for legal issues [7]. In addition, people were found to access help more commonly from non-legal practitioners including health or welfare practitioners which has implications for the quality and expertise of help [6,7]. Families experiencing adversity are among those more likely to have increased legal needs yet also face significant barriers accessing legal assistance [5].

HJPs embed legal support into health and community care teams to address unmet legal needs that can harm health [7,8]. HJPs aim to improve health by focusing on the upstream social and legal issues that impact health, upskilling health workforce to recognise and respond to legal issues and to improve access to legal support [7,9]. HJPs vary considerably, depending upon the service context, client needs, and the level of commitment to the HJP by health and legal partners. Partnerships can involve periodic legal help onsite, incorporating legal advisers into health teams, integrating legal support into care pathways and/or conducting joint clinics [7]. A common element across all types of HJP is a purposeful relationship between health and legal services to support shared clients with intersecting health and legal needs [7].

There is strong evidence that HJPs improve access to legal support for individuals and families experiencing adversity who would not otherwise access legal support [7]. Studies have demonstrated an increase in family income following HJPs intervention with a reduction in families avoiding medical care due to cost and greater adherence to medical treatment [7,10,11]. There is also evidence that HJPs improve individual mental health outcomes including a reduction in stress, depression and anxiety [7,9]. In addition, HJPs have positive effects on health practitioners with a reduction in stress, increased capacity to respond to legal issues and improved job satisfaction thus decreasing the risk of practitioners’ burnout [7,10,12].

However, despite the evidence for the effectiveness of HJPs, there is limited evidence for HJPs in community paediatric settings and no evidence about the barriers and facilitators to implementing a HJP within a community paediatric context in Australia. Implementing a HJP within an integrated health and social care child and family hub may improve outcomes for children by addressing adversity related to unmet legal issues within the family. Improving parent or primary caregiver (hereafter caregiver) access to legal support and providing ‘early intervention’ which is legal assistance and education prior to formal legal proceedings is an important first step [13]. Thus, we aimed to (1) evaluate the first 12 months of an HJP, assessing the feasibility and acceptability within a community paediatric context and (2) to explore both practitioner and caregiver experience of accessing legal support.

## Methods

This study is a mixed-method evaluation of newly established HJPs within the context of child and family health and social care hubs in two sites in Australia. The study was conducted in line with the National Statement on Ethical Conduct in Human Research [14]. Ethical approval was granted by The Royal Children’s Hospital Human Research Ethics Committee (HREC # 62866.7).

### Study Setting

Legal help was integrated into two community health services, one in Wyndham Vale, Victoria and one in Marrickville, New South Wales (NSW). This study was nested within a larger evaluation that was evaluating two integrated Child and Family Hubs (CFH) with a focus on identifying and responding to childhood adversity [15]. The codesign and implementation of the CFH have been discussed elsewhere with the scope of this paper limited to the evaluation of the HJP [16,17]. The community services are both located in metropolitan areas that have high population risk factors for adversity [18,19]. Across both sites, caregivers report high rates of adversity with approximately a third of 349 caregivers experiencing more than five adversities at the beginning of the CFH [20]. The HJP were developed over a 24 month period with the assistance of Health Justice Australia who initially approached legal services to assess their interest in developing a HJP within the CFH. Health Justice Australia then facilitated meetings between the legal services and researchers and developed an agreement to the scope of the partnership across the different sites. A legal needs assessment was conducted at both sites by Health Justice Australia 12 months prior to the CFH to tailor the HJP to meet the local need. Three separate legal organisations provided legal support across the two HJPs with two organisations providing support in Victoria and one organisation providing support in NSW. Both HJPs provided a range of legal assistance, including civil (e.g. credit and debt, tenancy, fines) and family law matters (e.g. family property, family violence, child protection). Legal services did not receive any additional funding in developing the HJP.

#### IPC Health Wyndham Vale, Victoria

Wyndham Vale is in the outer South-Western area of Greater Melbourne. IPC Health Wyndham Vale is a community and primary health service which has several services co-located including general practitioners, paediatricians, practice nurses, maternal child health nurses, allied health practitioners, oral health services and financial counselling. The Wyndham Vale HJP consisted of lawyers resourced by each partner organisation working onsite at IPC Health for one day on alternate weeks. Lawyers from both organisations attended regular monthly meetings with the health and social care practitioners from the CFH, providing training and support to practitioners to recognise legal needs and build awareness and connection to local legal services. Secondary consultations were also offered to practitioners. Legal support was offered either in person at IPC Health or remotely according to client preference. Lawyers were on site on the day that corresponded to the greatest number of CFH practitioners being present. Caregivers were referred for legal support by CFH practitioners. Both organisations provided legal support related to family law and family violence whereas referrals for financial, tenancy, civil matters or criminal matters were supported by one organisation. The two legal organisations met to develop a collaborative model for the HJP.

There was a turnover of legal staff over the study period. Of the original lawyers in the HJP, one lawyer was replaced due a change in role and the other two lawyers stepped back into a supervisory role of other lawyers from their organisation. Relationships were actively built over the duration of the study supported by regular monthly meetings.

#### Marrickville Community Health Centre, Marrickville, NSW

Marrickville is a suburb in the Inner-West of Sydney within the Sydney Local Health District. Marrickville Community Health Centre is an established community and allied health service and has child and family nurses, allied health practitioners, paediatricians, social workers, midwives, psychologists, mental health services and oral health services. The Marrickville HJP was a legal outreach service and consisted of a referral pathway a local community legal service which was located near to the community health centre. Lawyers were onsite at the Marrickville site one day on alternate weeks to promote the HJP with clients and practitioners as well as providing initial training to practitioners. Legal assistance was provided to clients either on site at Marrickville Community Health Centre as a “drop-in” service or at the local community legal service. Caregivers were referred to legal assistance by practitioners and were able to self-refer. Lawyers provided legal assistance on a range of matters including family law, youth legal services, tenancy/housing, domestic, sexual, and family violence, and general legal advice. This partnership took a longer time to establish and to secure the buy in of the health practitioners. A significant proportion of healthcare practitioners worked part time, and as a result not all were able to access legal outreach services on site. There were several changes to the HJP over the study period which affected implementation, including staff turnover in Marrickville Health Centre and change in research personnel which had a significant impact on the establishment of the HJP as the research staff were responsible for driving the partnership.

### Participants

Health, legal and social care practitioners who took part in the implementation of the CFH in either site, were invited to take part in an individual or group interview to explore their experience of the HJP.

Members of the research team recruited caregivers through purposeful sampling. Caregivers who had seen a lawyer as part of the HJP in either site were invited to take part in an individual interview to explore their experience. Caregivers at IPC Health Wyndham Vale were identified by the legal service. Caregivers at Marrickville Community Health Centre were recruited from service navigator clients who had engaged with the legal service. Caregivers who were enrolled as part of the broader study were also included if they had accessed help through the HJP in either site. Caregiver participants received a A$40 gift voucher for taking part. Informed consent was obtained prior to participation in an interview.

### Data Collection

#### Quantitative Data

Quantitative process data including referral data, demographic data, and outcomes of legal support was collected for all caregivers who accessed the legal service through the HJP over a 12-month period. Process data was collected from three months after the beginning of the CFH to allow for time for practitioners to receive education about the HJP and become comfortable to make referrals (June 2022–June 2023 for Wyndham Vale and August 2022–August 2023 for Marrickville). Client data was collected by the legal service however due to concerns about client confidentiality individual level data was then aggregated prior to sharing with the research team.

#### Practitioner Interviews

In depth semi-structured (n = 26) and group (n = 1) interviews were conducted with practitioners across the two sites. The interviews in Wyndham Vale were conducted by two members of the research team (SL, LNC) between March and May 2023 averaging 39 minutes (range 29 minutes–70 minutes). The interviews in Marrickville were conducted by two members of the research team (RB, HL) between October and November 2023 averaging 39 minutes (range 20 minutes–60 minutes). Interviews were conducted in person, via video conferencing platforms (Zoom or Microsoft Teams) or telephone according to participant preference. The interviews were recorded, transcribed, and edited for clarity and imported into NVivo 14 [21] for analysis.

#### Caregiver Interviews

In-depth semi-structured interviews were conducted with caregivers (n = 5 VIC and n = 2 NSW) between May and November 2023. The interviews in Victoria were conducted by one member of the research team (LC) averaging 68 minutes (range 29 minutes to 112 minutes) and the interviews in NSW were conducted by two members of the research team (RB, AP) averaging 16.5 minutes (13 minutes–20 minutes). Interviews were recorded and transcribed by Outscribe transcription service. Transcripts were edited for clarity and imported into NVivo 14 [21] for analysis.

### Qualitative Data Analysis

Interviews were analysed using thematic analysis. The analysis followed the six-step process as described by Braun and Clarke (2019); 1) familiarisation with the data, 2) coding, 3) generating initial themes, 4) renewing themes, 5) defining and renaming themes, and 6) writing up [22]. Coding of transcripts was inductive with keywords or phrases assigned a code using NVivo 14 [21]. The transcripts of the practitioners and the caregivers were analysed separately with all transcripts independently coded by members of the research team (SL, LC, AK, RB, HML). All transcripts in Victoria were dual-coded by two researchers (SL, LC, AK) whereas 50% of transcripts in NSW were dual-coded (RB, HML). Initial analysis from the two sites was conducted independently following the development of a shared code book. Codes and themes were developed through discussion across the wider research team with the lead author responsible for overarching theme development across both sites. Themes were tested against each transcript to ensure validity. The thematic analysis was then discussed and further developed with the legal services to improve the interpretation of the results.

### Reflexivity

Three members of the research team are medical doctors (SL, AP, HH). The other members include a psychology researcher with expertise in qualitative research (LC), a researcher in public health with experience in qualitative research (AK), a senior research officer (NW), a lived experience researcher (LNC), a clinical research nurse (WE), a research co-ordinator (RB), a senior research fellow with expertise in qualitative research (HML) and a research director with expertise in health justice partnerships (SF). Credibility of results was achieved through peer checking, triangulation of transcripts with interview notes and regular discussion as a research team and with legal services. Five members of the research team (SL, LNC, NW, RB, WE) were actively involved in implementation of the HJP across the two sites which provided deeper understanding of the challenges and barriers to execution of the HJP. As a lived experience researcher LNC was involved in the undertaking the research as well as the analysis and interpretation and writing of results.

## Results

### Quantitative Process Data

Across both sites, 40 clients were referred to the HJPs with 38 seen over the first 12 months as outlined in Table 1. There was a significant difference between the legal services that supplied legal support in the two CFHs. In Marrickville, there were only 3 caregivers seen in the first 12 months and all of these were self-referrals to the legal service. This contrasted with Wyndham Vale where 35 caregivers were referred by CFH practitioners across the two legal organisations. Most of the legal support was provided as advice (58%) however a third of caregivers received further support in the way of legal representation.

**Table 1:** Referrals for Legal Support (n = 40).


LEGAL SUPPORT		LEGAL SERVICE 1 N (%)	LEGAL SERVICE 2 N (%)	LEGAL SERVICE 3 N (%)	TOTAL N (%)

Total referrals to service		27	10^+^	3*	40

Total number of clients seen		27 (100)	8 (80)	3 (100)	38 (95)

Previously seen lawyer		2 (7)	5 (50)	0	7 (18)

Outcomes	Advice	13 (48)	6 (60)	3 (100)	22 (58)

	Representation	12 (44)	–	0	12 (32)

	Other	2 (7)	4 (40)	0	6 (16)


*All self-referrals ^+^One client referred 3 times.

Most of the caregivers seen in the HJP were female with just over a third born outside of Australia as seen in Table 2. Most caregivers had low incomes with only 5 caregivers having an income over $45,000 per annum. While most caregivers had 1 or 2 children, 18% of families had more than 4 children.

**Table 2:** Demographic characteristics of caregivers seeking legal support (n = 38).


DEMOGRAPHIC DATA OF PARENTS SEEKING SUPPORT		NUMBER OF CLIENTS (N)			TOTAL N (%)

		LEGAL SERVICE 1	LEGAL SERVICE 2	LEGAL SERVICE 3	

Age range	18–24 years	2	2	0	4 (11)

	25–34 years	13	2	0	15 (39)

	35–44 years	9	4	2	15 (39)

	45–54 years	3	0	1	4 (11)

Gender	Female	26	6	3	35 (92)

	Male	1	2	0	3 (8)

Ethnicity	Born outside Australia	12	1	1	14 (37)

	Aboriginal and Torre Strait Islander	0	–	0	0

	Unknown	–	7	–	7

Language	Language other than English at home	11	–	0	11 (29)

Household income*	$0–$18,200	9	–	2	11 (29)

	$18,201–$45,000	13	–	1	14 (37)

	$45,001–$120,000	5	–	0	5 (13)

	$120,001 and over	0	–	0	0

Number of children in household	1	7	5	2	14 (37)

	2	9	2	0	11 (29)

	3	4	–	0	4 (10)

	4 or more	5	1	1	7 (18)


*Income data missing from legal service 2.

**Figure 1:**
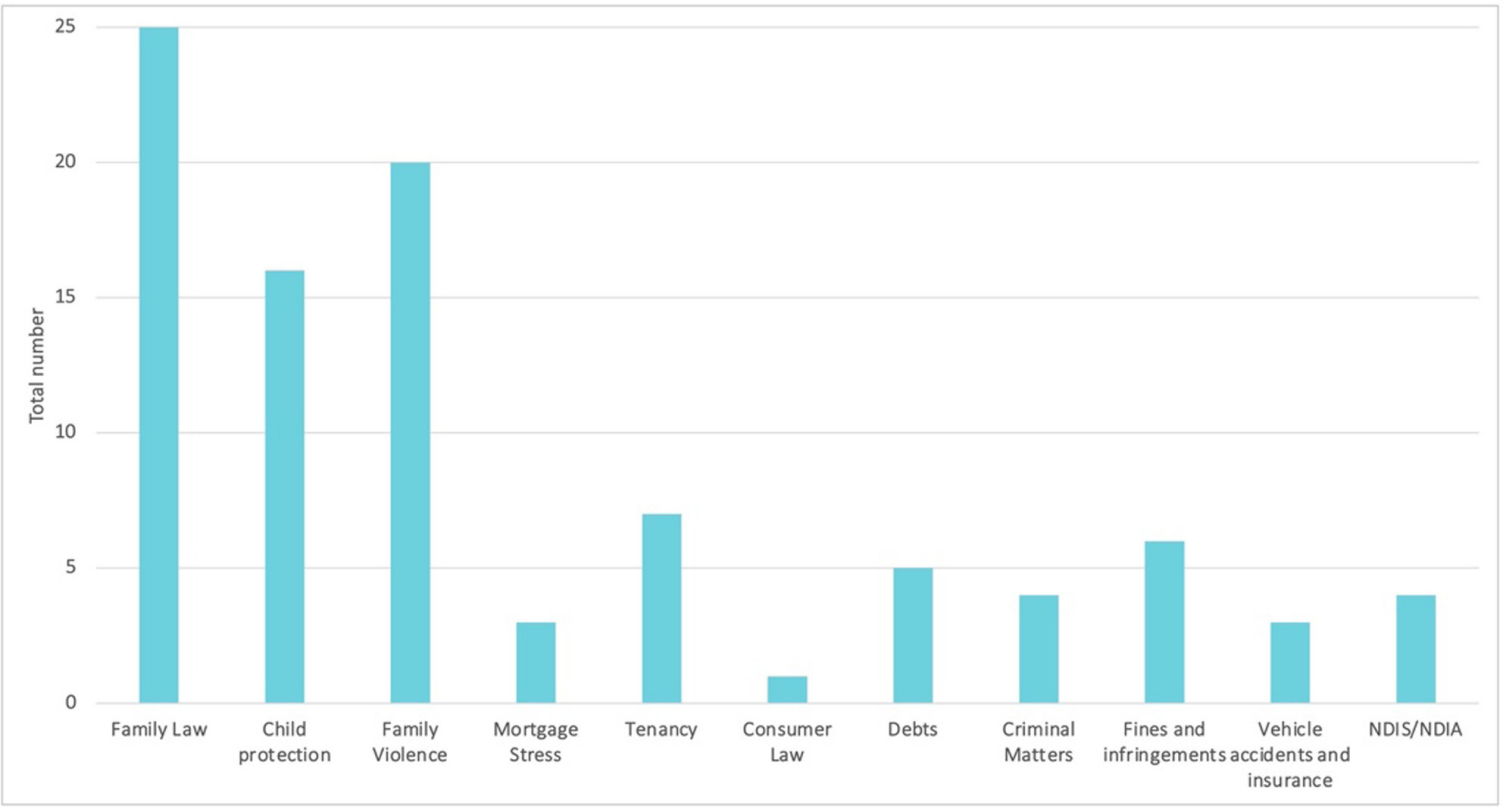
Legal Needs Identified.

There were a range of legal needs identified with family law (27%), child protection (17%) and family violence (21%) making up the majority as demonstrated in Figure 1. The range of legal needs highlights the importance of HJP providing both family law and civil law support. On average there were 2.5 legal needs per caregiver identified. Caregivers were generally referred for one legal issue and other issues were identified through the legal assistance.

### Qualitative Data

#### Practitioners

A total of 29 practitioners (n = 21 VIC and n = 8 NSW) participated in an in-depth semi-structured interview. Three nurse practitioners participated in a group interview while the remainder of the practitioners participated in an individual interview.

Six of the practitioners interviewed were lawyers working in the HJP. Due to the differences in settings, there were no primary care health practitioners interviewed at Marrickville. In addition, all the practitioners involved in the CFH at Wyndham Vale participated in an interview however, only 50% of CFH practitioners from Marrickville took part in an interview. Most of the practitioners interviewed were female with a range of age and experience as demonstrated in Table 3.

**Table 3:** CFH Practitioner characteristics (n = 29).


PRACTITIONER CHARACTERISTICS	N (%) N = 21 VIC	N (%) N = 8 NSW

Age		

18–24 years	1 (4.8)	–

25–34 years	3 (14.3)	4 (50)

35–44 years	9 (42.8)	2 (25)

45+ years	8 (38.0)	2 (25)

Number of years in role		

<2 years	2 (9.5)	4 (50)

3–5 years	6 (28.6)	1 (12.5)

6–10 years	6 (28.6)	1 (12.5)

>10 years	7 (33.3)	2 (25)

Service Provider Gender		

Male	3 (14.3)	1 (12.5)

Female	18 (85.7)	7 (87.5)

Role		

Paediatrician/Paediatric Fellow	3 (14.3)	1 (12.5)

General Practitioner	2 (9.5)	–

Nurse (Maternal Child Health Nurse, Practice Nurse)	6 (28.6)	2 (25)

Allied Health (Speech Pathologist, Dietician, OT)	2 (9.5)	2 (25)

Financial Councillor	1 (4.8)	–

Lawyer	5 (23.8)	1 (12.5)

Social worker	2 (9.5)	1 (12.5)


There were four themes identified from analysis of practitioner data across both sites (1) value of legal support, (2) improved understanding of legal support pathways, (3) connection increased practitioner confidence and (4) legal service underutilised.

##### Theme 1: Value of legal support

Practitioners recognised the value of having an HJP to provide quick and easy access to legal help but also to provide the appropriate legal support to families.

*“Right from the start when there was talk of access to lawyers, I was like, this is just fantastic, just to have easy access… And them, as part of the hub [CFH] just makes it that little bit less daunting for clients.” P9 VIC (social care practitioner)*

The HJPs made accessing information and making referrals easier for practitioners which increased the perceived value of the partnership.

*“I think it’s valuable, because I think there’s often a time where you can refer to legal quite conveniently, or have them next door or, you just have their email to ask questions or inquire with them about certain things.” P5 VIC (health practitioner)*

Practitioners acknowledged the importance of legal support as a key service to address unmet legal and social needs.

*“I would find it hard to do my job without having access to some sort of legal resource. Yeah, so they are vital. In fact, I would say in my lay view of Hubs [CFH] that you can’t have a hub without a lawyer or a legal service or without a financial counselling service.” P18 VIC (social care practitioner)*

Practitioners also recognised the value of HJP to not only provide easy access to legal help but also provide legal help early, thus preventing legal issues reaching crisis points.

*“That direct access to a lawyer, and a free lawyer, and I guess justice by extension. …The clients get free legal advice before an issue hopefully escalates, before a matter gets to court …they can have an advice session to try and resolve it and act as a prevention” P4 NSW (lawyer)*

While a range of practitioners across both sites recognised the value of the HJP, this did not equate to referrals being received from the range of practitioners; a few practitioners made most of the referrals.

##### Theme 2: Improved understanding of legal support pathways

Practitioners discussed the importance of HJPs in increasing knowledge of legal support pathways. Health practitioners reported having very limited understanding of the legal system and how to help families prior to involvement with the HJP.

*“Having them [legal service] come at the beginning of the process and say ‘We can actually help with this, and this, and this’ was brilliant. I had no idea of the scope of practice or that someone can just walk straight in the door and one of the team will speak to them” P3 NSW (health practitioner)*

This increased knowledge of the legal support options, increased practitioner confidence to ask families about legal issues and make referrals.

*“Because before the Hub [CFH] started, I didn’t know too much about the avenues to get legal support and … what it would involve, and it made it much easier to …ask questions around this, knowing that I have actually got something there that I can offer to the families.” P2 VIC (health practitioner)*

*“Maybe an increase in understanding in what we do, because, as you know, a lot of people don’t realize that these everyday life problems are also legal problems that they can get help with. So, I think that increased understanding probably led to more referrals as well, which was good.” P20 VIC (lawyer)*

Conversely, practitioners who reported limited understanding of the legal support options within the HJP were less likely to ask about legal issues, seeing this as a *“no go”* area and not something that they wanted to *“touch on or talk about”*. This lack of knowledge impacted practitioner confidence to identify legal issues as evidenced by *“if you don’t feel like there’s a service you can offer … then you’re not really going to pry or lean on that level of vulnerability” P18 NSW (health practitioner)*

##### Theme 3: Connection increased practitioner confidence

Knowing the lawyers in the CFH and being able to ask them for advice increased practitioner confidence to ask and respond to patients’ legal issues at Wyndham Vale.

*“Just to know that they [legal support] are there and to ask them those questions. Again it is good, and it was quite supportive and it gives you that confidence that you know, if you need something, you can always ask it.” P4 VIC (health practitioner)*

Colocation enabled practitioners to build trusting relationships with the legal team. Practitioners valued being able to ask for advice easily and quickly.

*“But also I think what was good, when we had the meeting if we needed to ask advice we could do that straightaway. That’s what I liked. That was really good. Because prior to that, you know, we could have referred to [legal service] but that would be it, we didn’t really have much to do with them.” P15 VIC (health practitioner)*

Practitioners were able to reflect on how they had changed their practice and were no longer afraid of identifying legal issues and were more likely to ask about legal issues rather than avoid it.

*“It’s the first time I have used a sort of lawyer … my first experience with it because I have always left it up to orange door*^1^ *or CPU*^2^ *to handle the legal side … It made it a lot easier to be able to approach a lawyer, and the lawyer the hub get is really good …. [it] has been a really good experience because I haven’t dealt with lawyers before and have just left it up to the client.” P21 VIC (health practitioner)*

Practitioners in Marrickville also recognised that the HJP made them feel “less anxious” to respond to legal issues and saw the value in having lawyers onsite to reduce the barriers to access for families.

*“And it doesn’t seem like far but if you’re a vulnerable mum with three kids and no car making it up the road is a whole world away. So, I actually think it does make a difference having them on site” P2 NSW (health practitioner)*

##### Theme 4: Legal service underutilised

Practitioners across both sites reported that they could utilise the legal support more but needed further support and capacity building.

Practitioners in Marrickville had limited understanding about the HJP and the legal support offered to families. This may have been related to the difficulties in establishing the HJP in Marrickville.

*“Everyone was confused and obviously I couldn’t go to my colleagues…and be like ‘what is this’ because they had no idea” P20 NSW (health practitioner)*

*“There’s a lack of knowledge that we [legal services] are there…they’re not always going to remember that a lawyer comes once a fortnight at this time…if the healthcare worker doesn’t know we’re there, the client’s not going to know we’re there unless they stumble upon us.” P4 NSW (lawyer)*

Practitioners across both sites were hesitant to ask about legal issues because they did not remember or failed to recognise legal needs or felt that this was out of their scope of practice.

*“Not saying that there’s not families that wouldn’t benefit, but I think that’s definitely an area that I just wouldn’t ask about and wouldn’t be aware of” P18 NSW (health practitioner)*

Sometimes not asking directly about legal needs was due to practitioner personal level of comfort about legal issues and fear of negative consequences such as impacting the relationship with the caregiver.

*“It was good, but I found it very confronting. Like how do I refer someone to a lawyer? Am I just like intruding in their household? Yeah, that’s how I felt about the lawyers, because … I’ve never dealt with a lawyer. I find it very intimidating.” P12 VIC (health practitioner)*

One of the main barriers identified to practitioners utilising the service is the time pressures of clinical work.

*“It was probably a time poor issue, but the …. core health practitioners, the GPs and the nurses that were on site, didn’t… really make any referrals into our services.” P20 VIC (lawyer)*

Practitioners also recognised that the availability of the legal support service also played a role in how well this was used by families.

*“It hasn’t really worked for me or my clients…they’re [lawyers] very approachable people…but the days didn’t overlap…People are generally time-poor, so even though they were available in there, I don’t think my particular clients made best use of the legal team” P3 NSW (health practitioner)*

#### Caregivers

Seven (of nine contacted) caregivers took part in an interview (5 in VIC and 2 in NSW). Several caregivers from Wyndham Vale consented to be contacted by the research team however declined to participate. Whereas in Marrickville, due to the small numbers of caregivers seen in the HJP only a small number were eligible and contacted for an interview.

Across both sites all the caregivers who participated in an interview were female and most were born in Australia and spoke English at home as seen in Table 4.

**Table 4:** Caregiver Interview Participant Characteristics (n = 7).


PARTICIPANT CHARACTERISTICS		N (%) N = 5 VIC	N (%) N = 2 NSW

Age (Median, range)		35 (26–46)	42 (39–45)

Gender	Female	5 (100)	2 (100)

Country of birth	Australia	3 (60)	2 (100)

	China	1 (20)	

	Togo	1 (20)	

Primary language spoken	English	3 (60)	2 (100)

	Other	2 (40)	

Aboriginal and Torres Strait Islander		0	0

Highest level of schooling	Year 10	1 (20)	1 (50)

	Year 12	1 (20)	1 (50)

	Trade or other certificate-level	2 (40)	

	Bachelor’s degree	1 (20)	

Number of children	1	1 (20)	

	2	3 (60)	1 (50)

	3 or more	1 (20)	1 (50)


There were three themes identified in the analysis of the caregiver interviews (1) Legal support empowers families, (2) Expectations of solving problems and more intensive support, and (3) Caregiver experience impacts engagement.

##### Theme 1: Legal support empowers families

Legal support can empower families through providing advice and assistance. Caregivers reported feeling more confident to take the next step which led to a reduction in caregiver stress.

*“She just explained everything really clearly to me and gave me an outcome, pretty much of what I was wanting to find out, and she told me all the reasons behind it and everything like that, so I was happy with that.” Caregiver1 VIC*

*“Just help us with the next step and [to feel] more calm and we know a little bit of what’s happening… With her help, we just feel more confident… More confident in going through to the next steps.” Caregiver2 VIC*

Even when the advice was not successful in achieving the outcome the caregiver wanted, just having a clear idea of what to do instilled confidence.

*“The part where they gave me steps for me to follow, like, we think you should do this, you should do that. And I did try. It didn’t turn out at the end, but I still followed their steps …. They gave me ideas of what to do…because they’re the professionals, so I was listening and taking their advice, so I would say I was confident.” Caregiver4 VIC*

##### Theme 2: Expectations of solving problems and more intensive support

With the offer of legal help through the HJP, caregivers reported having expectations that the legal support would help solve their problems. For some, getting advice was seen as not enough and this unmet expectation led to a negative perception of the legal support. “*And I don’t feel my problem solved, that’s the thing.” Caregiver5 VIC*

*“I didn’t feel like I resolved anything…There was no solution. There was no outcome from that.” Caregiver4 VIC*

Caregivers wanted to have more active help and multiple appointments with an expectation of more intensive support.

*“It was helpful, but not enough yet. I still need more.” Caregiver2 VIC*

##### Theme 3: Caregiver experience impacts engagement

Caregivers’ experience of legal support, both past and present, impacted on their ongoing engagement. For some, having previous negative experiences of seeking legal support influenced how they experienced help from HJP lawyers in the CFH. Some caregivers felt let down and struggled to trust the HJP lawyers.

*“Then I suppose if you [lawyers] trying to help people, you have the knowledge, you’re here to help, I don’t have to worry, but by the meantime I still worried all the time. I don’t have the trust. I don’t feel trust, that’s the thing.” Caregiver5 VIC*

Caregivers reflected on their previous experience of difficulty accessing legal support prior to the HJP due to restrictive eligibility criteria for funded services which led to seeking legal support in the private sector. Affordability and eligibility of legal support was a key determinant of caregiver engagement. Caregivers were concerned about engaging with legal support *“because if it’s too expensive, I can’t afford to” Caregiver1 VIC*.

## Discussion

This mixed methods study is the first study to evaluate a HJP within a community paediatric context in Australia and to explore both practitioner and caregiver experience of accessing legal support. The HJP increased access to legal support for vulnerable families with 85% of caregivers who accessed legal support through the CFH having no previous legal assistance. Caregivers had multiple legal needs across a broad range of legal problems affecting their families, with family law, child protection and family violence being the most common legal needs. The HJP was found to be acceptable within a community paediatric context to both practitioners and caregivers, however, feasibility was affected by lack of funding and difficulties in establishing the relationships across the two sites.

Practitioners reported feeling more confident to respond to legal issues and valued the ease at which they could get legal support for their families. This improvement in confidence is supported in the literature with practitioners across a range of HJPs reporting improved confidence to address social determinants of health during clinical encounters [7,10]. Practitioners across several HJPs have reported the value of a HJP in being able to provide better and more efficient care [10,23,24]. Practitioners are more likely to feel that they can respond to legal needs if they have a relationship with the lawyers providing the legal support. The relationships built between HJP lawyers and health practitioners in our study were critical to improve practitioner confidence and referrals and were supported with lawyers being co-located and meeting regularly with practitioners. Co-location has been identified as a key feature of successful a HJP, facilitating “mutual trust” between health practitioners and lawyers and avoids the HJP becoming just an “afterthought” [23,24]. The ability to “just walk down the hall” (pg7) was seen as fundamental in building good relationships [24]. However, relationships take time to build and may impact health practitioner income depending on practice funding model. The potential benefits of decreased practitioner stress, and greater clinical efficiency may offset this challenge [7,12].

Despite the recognition of the value of the HJP, practitioners across both sites reported it was underutilised in this establishment phase. Marrickville had very low numbers of caregivers accessing legal support through the CFH despite having a similar baseline adversity to the Wyndham Vale site [20]. There were no referrals to the Marrickville HJP lawyers during the 12-month study period by the CFH practitioners. There are several factors that influenced this. Most of the essential work to make the CFH practitioners aware of the HJP, and to build their knowledge, skills, confidence, and trust to refer clients to the lawyers, only took place later in the study period. Contributing to this was a significant change of staff at the Marrickville HJP with the research team and lawyers all changing over the 12-month study period. Not having the lawyer join regular meetings limited the opportunities to build relationships and offset the effect of staff turnover. This contrasted with Wyndham Vale HJP where there was a monthly meeting of all CFH practitioners and HJP lawyers which built relationships across the HJP. However, most of the referrals at Wyndham Vale were from only a few practitioners. This may reflect the time it takes to build confidence and feeling of comfort of health practitioners to ask about and respond to legal issues and the need for more training and support of health practitioners in recognising legal needs [24]. Improving HJP implementation requires robust education and training of practitioners to increase practitioner comfort and confidence to respond to legal issues [25]. It is critical to support future HJP investments by building relationships and developing shared processes across both health and legal if we are to see improved outcomes from HJP.

HJP is a strategy to increase the range of services and expertise, beyond only that of health practitioners, available in a single accessible location to support families experiencing intersecting health and legal issues. Recognising how different types of legal issues cluster, caregivers may need a *range* of legal expertise, across family, civil and criminal law. In Wyndham Vale, one legal service provided advice and assistance for a broad range of legal needs including civil law (e.g. credit and debt, tenancy, fines), criminal and family law issues, while the other focused on providing advice and representation for family law issues only. HJP are more successful if a range of services are offered as exemplified in a set of pilot HJPs supporting women at risk of family violence in the Australian Capital Territory. Assistance was provided not only for family violence issues, but also parenting, child protection, debt, and housing issues [26]. Providing a range of legal and non-legal support in the HJP was instrumental in improving outcomes for clients [25,26].

However, legal need is not just about the range of legal issues covered, but the level of support that clients may need to address issues ranging from advice only, to assistance or to full legal representation. Caregivers reported that they want more than just advice and had an expectation that legal support could solve their issues. It is important to build into future HJP adequate funding to enable lawyers to provide the level and range of assistance services that is appropriate to the needs of the clients [27]. Equally, it is important to manage caregiver expectations of what can be progressed or resolved through legal action and what cannot [27].

The low or no cost aspect of HJPs improve caregivers’ confidence in being able to access the legal system [10]. This was an important finding in our study; caregivers reported that they would not have accessed legal support outside of the HJP due to the expense. In addition, trusting relationships and warm referrals from health practitioners facilitate access to legal support. In a study of providing legal advice within general practice in the UK, 66% of families would not have sought assistance unless they had been referred by their health practitioner [28]. Not only do trusted relationships with health practitioners improve caregiver engagement with HJP lawyers but having lawyers physically integrated into health services also improves engagement [23]. Caregivers are more likely to first present to non-legal practitioners with social and legal needs rather than legal practitioners [29]. However previous experiences of the law can colour people’s perceptions of legal services and processes. This was seen with some of the caregivers in our study who had previously had negative experience of legal assistance, which in turn impacted their perception of the HJP [30]. Building trust with caregivers is an important step in improving access to legal assistance, and health practitioners may be well placed to facilitate improved trust with lawyers through a HJP.

### Strengths and Limitations

This is the first study to explore the acceptability and feasibility of a HJP within a paediatric community health context in Australia. We had broad participation across a range of health, legal and social care practitioners and explored the experience of both caregivers and practitioners in accessing legal support.

This study has some limitations. First, the study was undertaken during the establishment phase of the HJPs, while relationships and capability building was still underway and the results do not necessarily reflect a fully funded, established and embedded HJP.

Second, as an unfunded HJP, the lawyers were onsite once a week or fortnight which impacted on the time available to build engagement between practitioners. In other established HJPs, lawyers are embedded 2–5 days a week, which supports much higher visibility, stronger relationships and integration to the health site, as well as resourcing to assist more clients.

In addition, there were significant differences between the two sites which means the results are not comparable. However, the differences between the sites are useful in highlighting the important learnings for future HJP as discussed.

Third, there were a small number of caregivers who participated in an interview and their previous experiences of seeking legal support may have biased their experience of the HJP in the CFH. This small number may reflect the social complexity of the families or may be related to stigma of needing legal help. In addition, the caregivers in our study were all female and majority English speaking so further work is needed to explore the experience of a broader range of caregivers in accessing legal support.

Finally, this study explored the HJP within the context of a CFH which was implemented just after the Covid-19 pandemic. Over the study period there were periods of high infection rate which impacted on the medical workforce through increased workload which may have impacted on referral rates.

## Conclusions

We have demonstrated that HJP improved access to legal help for families experiencing adversity by building a referral pathway through health services they were already accessing. This provides the opportunity for early intervention for legal issues that are affecting caregivers and their children and can improve the response to childhood adversity.

With investment from both partner services, HJPs can enhance health practitioner confidence to address legal needs through building relationships with the HJP lawyers. However, HJPs take time and investment in relationships, resourcing, and joint processes to establish. Health and legal services seeking to work in this way must factor this foundational work into their funding advocacy and applications, stakeholder engagement and collaborative service design in an ongoing iterative fashion help to embed and create lasting and functional HJPs.

## Footnotes

The Orange Door is a state government service that provides help for people experiencing family violence, or who need assistance with the care and wellbeing of children and young people.

CPU = child protection unit.
